# Longitudinal study of antibody response following SARS-CoV-2 vaccination with Pfizer-BioNTech BNT162b2

**DOI:** 10.1515/almed-2023-0052

**Published:** 2023-08-29

**Authors:** Leandra J. Robles Navas, Juan J. Ortega Huete, María L. Juliá Sanchis, Jorge S. Bravo Miró, Serezade Cervera Sánchez, Ricardo Molina Gasset

**Affiliations:** Internal Resident, Service of Clinical Analysis, Hospital Virgen de los Lirios, Alcoy, Spain; Specialising in Medical Biology, Service of Clinical Analysis, Hospital Virgen de los Lirios, Alcoy, Spain; Service of Clinical Analysis, Hospital Virgen de los Lirios, Alcoy, Spain; Nurse, Preventive Medicine Service, Hospital Virgen de los Lirios, Alcoy, Spain; Head of Clinical Analysis Service, Hospital Virgen de los Lirios, Alcoy, Spain

**Keywords:** antibodies, COVID-19, immunogenicity, vaccine

## Abstract

**Objectives:**

To assess the immunity of healthcare and non-healthcare workers in the Alcoy health area (Spain) after completing three doses of the Pfizer-BioNTech vaccine and how it relates with individual factors.

**Methods:**

We conducted a prospective, observational, longitudinal, analytical study to observe immunogenicity in healthcare and non-healthcare workers at Virgen de los Lirios Hospital following administration of three doses of the Pfizer-BioNTech vaccine. Anti-SARS-CoV-2 spike protein IgG antibody concentrations were determined, while COVID-19 infections were identified by qualitative detection of IgG against the nucleocapsid protein. Samples from volunteers were analyzed 15 days, 3 months, and 6 months after receiving the second vaccine dose, and 12 months after receiving the third (booster) dose. A questionnaire was used to collect demographic data, risk factors, and use of immunosuppressive drugs. Statistical analyses were performed with SPSS software, and mean antibody levels over time were compared using ANOVA.

**Results:**

Participation was 99.5 % (199/200). The IgG concentration was higher in men and in obese individuals. No significant changes were noted in factors such as smoking.

**Conclusions:**

All participants developed humoral immunity, except for three subjects who were receiving immunosuppressive treatment. Participants with previous COVID-19 infection had higher antibody levels.

## Introduction

Severe acute respiratory syndrome coronavirus 2 (SARS-CoV-2) is a virus of the *Coronaviridae* family that causes coronavirus disease 2019 (COVID-19). It was first detected in December 2019 in the city of Wuhan in the Hubei Province [1] of China and, due to the rapid increase in cases and deaths, it was declared a pandemic by the World Health Organization on 11 March 2020 [2].

Although a series of measures were implemented, the spread of the virus was continuous and sustained, and its impact on the world forced the scientific community to develop effective vaccines against this pathogen as a matter of priority.

The proposed approach was to target the spike (S) protein [3] of the virus–previously considered the best antigen to induce neutralizing antibodies against other members of the family–as the antigen of choice. As such, it has been the protein primarily used to develop COVID-19 vaccines.

mRNA-based vaccines [4] have the advantage that they are easy to produce and act quickly once inside the cell, facilitated by their coupling and stability in encapsulated lipid nanoparticles that express the S protein, thus inducing a good humoral and cellular immune response. This method was chosen by Moderna and Pfizer/BioNTech.

Our aim, therefore, was to analyze the response to the Pfizer-BioNTech mRNA vaccine in a cohort of healthcare and non-healthcare workers, including those with or without previous COVID-19 infection.

## Materials and methods

We conducted a prospective, observational, longitudinal, analytical study to observe immunogenicity after administering three doses of the Pfizer-BioNTech vaccine to healthcare and non-healthcare workers at Virgen de Los Lirios Hospital (Alcoy, Alicante province, Spain). The determined parameter was IgG antibodies against the SARS-CoV-2 S protein. Samples from volunteers were measured 15 days, 3 months, and 6 months after receiving the second dose of the vaccine, and 12 months after receiving the third (booster) dose of the vaccine. The aim was to study the immunogenicity of the vaccine in volunteers based on two main variables: age and sex. Other important information such as body mass index (BMI), smoking history, medical history, and previous COVID-19 infection (and when, in case of previous infection) were obtained to complete the study and to determine whether other relevant conclusions could be drawn.

### Sample size

A total of 199 healthcare and non-healthcare workers from the Alcoy healthcare area (Alicante province, Spain) who had received two doses of the Pfizer-BioNTech vaccine were randomly selected and stratified into two groups according to age: <40 years and >40 years. Stratification was performed using an Excel sheet that included no sensitive patient data, in which all participants were classified by age; 107 subjects were then randomly selected from the group aged <40 years and 92 subjects from the group aged >40 years. Once they had been selected, a list was drawn up with the telephone numbers of the selected subjects and they were informed of the possibility of participating in the study.

### Post-vaccination questionnaire

The subjects then went to the hospital, where they were given a patient information sheet and informed consent form. Those who agreed to take part in the study were given a test request form, identified only by an assigned number, and an appointment was made to have blood samples drawn. They were administered a survey identified by their assigned number in order to preserve data confidentiality. The survey included various items such as age, sex, smoking habits, previous history of COVID-19 (if any), presence of any disease, and medication use. Using this information, together with the data obtained in the laboratory tests, we developed a database for analysis.

### Determination of anti-SARS-COV-2 antibodies

Antibody levels were determined by high-throughput chemiluminescent microparticle immunoassay (CMIA) on the Abbott Laboratories Alinity i^®^ platform (Abbott, Abbott Park, IL, USA) in the Department of Laboratory Medicine at Virgen de los Lirios Hospital, Alcoy. The assays used were quantitative detection of IgG antibodies against the S protein, with a sensitivity of 97 % and specificity of 99.6 %. Subjects were considered to have antibodies when the result was >49 AU/mL (arbitrary units [AU]/mL).

### Statistical analysis

As mentioned above, the clinical information collected and the laboratory results obtained were used to develop a database, from which the study results and conclusions were drawn. The statistical analysis itself was carried out using SPSS 15.0 software (IBM Corporation, Armonk, NY, USA).

Given the sample selected, normal distribution can be assumed, so parametric tests were used when performing the analysis. The means of the antibodies over time were compared using analysis of variance (ANOVA) with respect to age, sex, BMI, and smoking habit.

### Ethics committee approval

The study was approved by the Hospital General Universitario de Elda (Alicante province, Spain) Ethics Committee for Research with Medicinal Products.

## Results

Initial participation was 99.5 %: 107 workers aged <40 years and 92 workers aged >40 years, with mean age 42.62 years. Regarding lifestyles that initially suggest a lower prevalence of obesity and smoking in health-related occupations, 49 % were never-smokers, 54 % of subjects had a normal BMI, and only 8.5 % reported previous COVID-19 infection. Table 1 shows the characteristics of the population.

**Table 1:** General characteristics of the study sample.

Variable	Mean (95 % C.I.)	Minimum–maximum
Age, years	42.62 (38.92–42.15)	22–64

**Variables**	**Category**	**n (%)**

Sex	Men	100 (50.3 %)
	Women	99 (49.7 %)
Age	<40 years	107 (53.8 %)
	>40 years	92 (46.2 %)
Smoking habit	Never smoker	96 (48.2 %)
	Former smoker	46 (23.1 %)
	Active smoker	57 (28.6 %)
Body mass index	Normal (18.5–24.9)	107 (53.7 %)
	Overweight (25–29.9)	67 (33.6 %)
	Obesity class I and II (30–40)	25 (12.5 %)

Table 2 shows the distribution of the workers, correlating the prevalence of antibodies against SARS-CoV-2 (AU/mL) in relation to age. In healthcare and non-healthcare workers over 40 years old, the mean IgG antibody concentration was higher than in those under 40 years old, although the difference was not significant. The results obtained show that 98.5 % of the participants presented IgG antibodies against the S protein.

**Table 2:** Analysis of concentration of IgG antibodies against COVID-19 S protein and age.

Variables	Category age	Mean quantitative IgG AU/mL	95 % C.I.	p-Value^a^
15 days after receiving the 2nd vaccine dose	<40 years	19,251	17,086–21,415	0.356
	>40 years	17,363	13,687–21,039	
3 months after receiving the 2nd vaccine dose	<40 years	3,976	3,352–4,599	0.731
	>40 years	4,403	1,706–7,101	
6 months after receiving the 2nd vaccine dose	<40 years	2,021	816–3,226	0.848
	>40 years	2,196	842–3,550	
12 months after receiving the 3rd vaccine dose	<40 years	31,587	26,738–36,435	0.388
	>40 years	35,077	28,485–41,670	

^a^p-Value<0.05 was considered significant.

Table 3 shows the distribution of workers, correlating the prevalence of antibodies against SARS-CoV-2 (AU/mL) in relation to sex. The mean level of anti-S protein IgG antibodies at completion of the vaccination schedule was 33,166.35 AU/mL (95 % C.I. 29,205.16–37,127.53). Throughout the study, men were found to have a higher mean IgG immunity to the S protein than women, which was significant at 12 months after the third vaccine dose.

**Table 3:** Analysis of concentration of IgG antibodies against COVID-19 S protein and sex.

Variables	Category sex	Mean quantitative IgG AU/mL	95 % C.I.	p-Value^a^
15 days after receiving the 2nd vaccine dose	Men	18,506	15,143–21,869	0.942
	Women	18,358	16,179–20,538	
3 months after receiving the 2nd vaccine dose	Men	4,602	2,253–6,950	0.472
	Women	3,717	3,098–4,335	
6 months after receiving the 2nd vaccine dose	Men	2,507	902–4,112	0.369
	Women	1,693	873–2,512	
12 months after receiving the 3rd vaccine dose	Men	39,801	33,219–46,383	0.001
	Women	26,751	22,539–30,962	

^a^p-Value<0.05 was considered significant.

Table 4 shows the distribution of anti-S protein IgG values over time in relation to BMI.

**Table 4:** Analysis of concentration of IgG antibodies against COVID-19 S protein and BMI.

Variables	Category BMI	Mean quantitative IgG AU/mL	95 % C.I.	p-Value^a^
15 days after receiving the 2nd vaccine dose	Normal	17,225	15,202–19,248	0.381
	Overweight	19,345	14,634–24,055	
	Obesity	21,161	16,424–25,898	
3 months after receiving the 2nd vaccine dose	Normal	3,687	3,057–4,317	0.495
	Overweight	5,185	1,679–8,691	
	Obesity	3,460	2,664–4,257	
6 months after receiving the 2nd vaccine dose	Normal	2,070	804–3,337	0.892
	Overweight	2,327	647–4,007	
	Obesity	1,610	160–3,059	
12 months after receiving the 3rd dose of the vaccine	Normal	30,689	25,195–36,183	0.050
	Overweight	32,489	26,553–38,424	
	Obesity	45,856	31,452–60,259	

^a^p-Value<0.05 was considered significant.

Although there were differences in antibody generation between participants with normal BMI and those with overweight or obesity, these were not significant, except at 12 months after the third dose, when a p-value of 0.05 was found and considered on the borderline of statistical significance. After performing the corresponding *post hoc* tests (Table 5), differences on the borderline of statistical significance were observed between subjects with normal BMI and those with obesity.

**Table 5:** *Post hoc* tests on concentration of IgG antibodies against COVID-19 S protein and BMI.

Variable	Category BMI	Mean quantitative IgG AU/mL	95 % C.I.	Mean difference	p-Value^a^
12 months after receiving the 3rd vaccine dose	Normal	30,689	25,195–36,183	−1,780	1.000
	Overweight	32,489	26,553–38,424		
	Normal	30,689	25,195–36,182	−15,167	0.050
	Obesity	45,856	31,452–60,259		
	Overweight	32,489	26,553–38,424	−13,367	1.340
	Obesity	45,856	31,452–60,259		

^a^*Post hoc* test, p-value<0.05 was considered significant.

Table 6 shows the production of IgG antibodies by the vaccine in relation to smoking. No statistically significant result was observed throughout the study, but the considerable increase in the mean quantitative IgG in former smokers with respect to never smokers and active smokers should be noted.

**Table 6:** Analysis of concentration of IgG antibodies against COVID-19 S protein and smoking.

Variables	Category smoking habit	Mean quantitative IgG AU/mL	95 % C.I.	p-Value^a^
15 days after receiving the 2nd vaccine dose	Never smoker	18,674	16,445–20,902	0.385
	Former smoker	20,378	13,643–27,113	
	Active smoker	16,501	13,752–19,250	
3 months after receiving the 2nd vaccine dose	Never smoker	4,077	3,348–4,806	0.239
	Former smoker	5,854	765–10,942	
	Active smoker	2,969	2,333–3,606	
6 months after receiving the 2nd vaccine dose	Never smoker	2,310	877–3,743	0.446
	Former smoker	3,173	762–5,585	
	Active smoker	825	592–1,057	
12 months after receiving the 3rd vaccine dose	Never smoker	31,795	25,989–37,601	0.521
	Former smoker	40,896	31,218–50,574	
	Active smoker	28,934	22,989–34,880	

^a^p-Value<0.05 was considered significant.

## Discussion

Our results show that 98.5 % of the participants presented IgG antibodies against the S protein, suggesting that the vaccine confers very high protection in high-risk personnel. Only three people failed to generate antibodies, all of whom were receiving immunosuppressive treatment. Smoking has been described as a possible severity factor for COVID-19 [5, 6], but few studies have been published linking smoking to a lower vaccine response. In this study, former smokers generated more antibodies than never smokers and active smokers, although the difference was not statistically significant.

It has generally been observed in COVID-19 patients that a higher BMI correlates with a higher level of SARS-CoV-2 antibodies [7]. Pellini et al. [8], studying only vaccinated individuals, found no association between BMI and IgG antibodies levels, while Watanabe et al. [9] found that subjects with higher abdominal obesity had lower IgG antibodies levels. In our study, we observed that patients with obesity only obtained a higher antibody concentration when they had received three doses of the vaccine. Studies with larger samples are needed to confirm these findings.

Given the efficacy of the vaccines that have been used, it is not currently recommended to check the immune response by measuring antibody levels [9]. This approach is supported by our results. Our study has the following strengths: 1) it was based on a representative sample of all hospital staff and had a very high participation rate and 2) the serological tests used have high sensitivity and specificity. Limitations may include the following: 1) the sample size was too small to draw definitive conclusions on response in the production of IgG antibodies against the S protein after vaccination; 2) our results cannot be extrapolated to the general population due to both age and the increased risk of exposure to the disease; and 3) since it is a new virus, there are many issues that are still unknown and, therefore, there could be other factors influencing the results that have not been analyzed. Follow-up of this cohort of vaccinated healthcare and non-healthcare workers should be considered, in order understand more precisely the dynamics of the immunity conferred by the vaccine and its persistence over time with a view to subsequent revaccinations. All participants developed a humoral immune response, except three, who were immunosuppressed. The variables related to higher antibody levels were being male, having a BMI >30 and having quit smoking.
